# High-Throughput Sequencing of the Expressed Torafugu (*Takifugu rubripes*) Antibody Sequences Distinguishes IgM and IgT Repertoires and Reveals Evidence of Convergent Evolution

**DOI:** 10.3389/fimmu.2018.00251

**Published:** 2018-02-21

**Authors:** Xi Fu, Jianqiang Sun, Engkong Tan, Kentaro Shimizu, Md Shaheed Reza, Shugo Watabe, Shuichi Asakawa

**Affiliations:** ^1^State Key Laboratory of Biotherapy, West China Hospital, Collaborative Innovation Center and Sichuan University, Chengdu, China; ^2^Laboratory of Aquatic Molecular Biology and Biotechnology, Department of Aquatic Bioscience, Graduate School of Agricultural and Life Sciences, The University of Tokyo, Tokyo, Japan; ^3^Bioinformational Engineering Laboratory, Department of Biotechnology, Graduate School of Agricultural and Life Sciences, The University of Tokyo, Tokyo, Japan; ^4^School of Marine Biosciences, Kitasato University, Kanagawa, Japan; ^5^Department of Fisheries Technology, Bangladesh Agricultural University, Mymensingh, Bangladesh

**Keywords:** antibody repertoire, heavy (H) chain complementarity-determining region 3, teleost fish, convergent evolution, IgM, IgT

## Abstract

B-cell antigen receptor (BCR) or antibody diversity arises from somatic recombination of immunoglobulin (Ig) gene segments and is concentrated within the Ig heavy (H) chain complementarity-determining region 3 (CDR-H3). We performed high-throughput sequencing of the expressed antibody heavy-chain repertoire from adult torafugu. We found that torafugu use between 70 and 82% of all possible V (variable), D (diversity), and J (joining) gene segment combinations and that they share a similar frequency distribution of these VDJ combinations. The CDR-H3 sequence repertoire observed in individuals is biased with the preferential use of a small number of VDJ, dominated by sequences containing inserted nucleotides. We uncovered the common CDR-H3 amino-acid (aa) sequences shared by individuals. Common CDR-H3 sequences feature highly convergent nucleic-acid recombination compared with private ones. Finally, we observed differences in repertoires between IgM and IgT, including the unequal usage frequencies of V gene segment and the biased number of nucleotide insertion/deletion at VDJ junction regions that leads to distinct distributions of CDR-H3 lengths.

## Introduction

The adaptive immune system (AIS) is fundamentally reliant on a highly diverse set of antigen receptors. These receptors are generated through somatic recombination of tandemly arranged variable (V), diversity (D), and joining (J) segments of the B-cell antigen receptor [BCR, the membrane-bound form of antibodies or immunoglobulins (Igs)] and T-cell receptor (TCR) genes, and the insertion and deletion of nucleotides (nts) at the junctions between ligated segments (1). The variety of generated antibody or Ig repertoires is required to recognize and bind various antigens (pathogens). The majority of diversity in the naïve Ig repertoire lies within the heavy (H) chain complementarity-determining region 3 (CDR-H3), which consists of the VDJ recombination junctions, and thus is the most diverse component and a major determinant of Ig specificity (2, 3).

Estimates of Ig diversity have been previously surveyed in several species (4–9). For the human BCRs, for example, the potential diversity of Ig molecules is estimated to be >10^13^ (9), while this number exceeds the total number of B cells in the human body (approximately 1–2 × 10^11^) (10). This excess of potential Ig diversity leads to the expectation that different individuals could seldom share the same segment rearrangement. Nevertheless, several studies have demonstrated overlap among the Ig repertoires of different individuals occurring, i.e., in the naïve human and zebrafish IgH repertoires (8, 11), and in the B-cell responses to virus infection in the rainbow trout (12).

Teleost fish are the most primitive bony vertebrates that contain Igs (13). Like humans, teleost fish have Ig gene rearrangement, junctional diversity during recombination, and somatic hypermutation (13, 14). It is also well known that smaller model organisms that contain fewer cells in total and obviously fewer immune cells can provide a better starting point from which to obtain a better coverage of the immune repertoire (15). In this regard, we chose to characterize the antibody repertoire of torafugu (*Takifugu rubripes*), a species that possesses the shortest genome of any known vertebrate, yet contains gene characteristics similar to those in the human genome (16).

We developed an analytical tool (PyDAIR, https://github.com/biunit/PyDAIR) to investigate the quiescent state of the torafugu immune system. Using the Illumina Miseq high-throughput sequencing platform allowed 12 million expressed antibody sequences from three healthy adult torafugu. We found evidence that the repertoire of individual fish is highly biased toward sequences with specific VDJ combinations, in addition to the convergent evolution of CDR-H3 sequences in both IgM and IgT. We observed distinct repertoires for IgM and IgT, respectively, which may suggest different mechanisms of diversity creation in fish naïve immune system.

## Results

We characterized the expressed IgH repertoire from healthy adult torafugu. To this end, we used a high-throughput sequencing approach that determines CDR-H3 profiles for both IgM and IgT, based on PCRs between Ig V and C domains. More than 8 million CDR-H3 amino-acid (aa) sequences were captured in our data set. We found that on an average, 180,062 and 24,933 distinct CDR-H3 clusters were presented in the naïve IgM and IgT repertoires, respectively. Each consensus (merged) read was assigned to a V and J gene by alignment to the germline references with success rates of ~94% for IgM and of ~98% for IgT (Table 1). Identifiable VJ reads were used for subsequent analysis. On an average, Dμ (Dm) segments were assigned to ~14% of the VJm reads and Dτ (Dt) to ~40% of VJt reads (Table 1); many of the unidentifiable cases had D regions deleted.

**Table 1:** Summary of consensus reads assigned to each V, D, and J gene segment measured for both IgM and IgT groups.

Fish	1	2	3
Total IgM reads	1,798,717	2,337,015	2,029,082
Identifiable VJm	1,684,225	2,215,821	1,915,237
Identifiable VDmJm	251,746	320,638	244,172
VJm assignment rate	0.936	0.948	0.943
VDmJm assignment rate	0.149	0.144	0.127
Total IgT reads	2,160,668	1,516,736	1,905,817
Identifiable VJt	2,096,641	1,483,036	1,876,739
Identifiable VDtJt	1,462,606	545,230	306,943
VJt assignment rate	0.970	0.977	0.984
VDtJt assignment rate	0.697	0.367	0.163

While the use of a PCR step before sequencing could potentially introduce bias in the inferred relative abundance of the sequences, due to differences in the efficiency of PCR amplification using different sets of primers. It is noteworthy that in the present study, the estimation of relative usage of V, D, and J gene segments was based on the hypothesis that there is no significant difference of PCR efficiency between primer combinations.

### Highly Biased VDJ Usage in the Naïve IgM and IgT Repertoires

#### IgM

**Figure 1:**
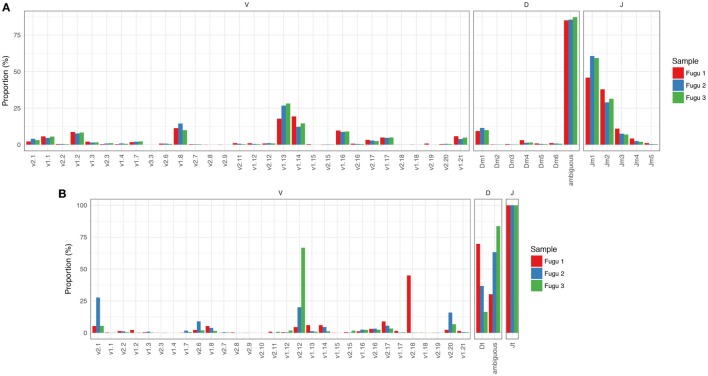
Overall IgM **(A)** and IgT **(B)** repertoires across the three torafugu. Non-uniform usages of V, D, and J gene segments were observed. VDJ gene segments are presented in 5′ to 3′ order as in genomic arrangement.

Focused on the naïve IgM repertoire, the results showed that frequencies at which specific V, D, and J gene segments were used were roughly consistent among individuals. Of the 32 potentially functional V genes, we found that 30 V genes from the three known IGHV groups were used by IgM, suggesting that most of the potential IgM repertoire was expressed in healthy fish. A preference for the usage of IGHV1 family sequences was evident, with V1.13, V1.14, and V1.8 occurring most frequently. The top five V genes accounted for over 65% of all VJm reads. Jm usage could vary by more than 100-fold, with Jm1 and Jm2 being favored, ranging from 55% for Jm1 to 0.6% for Jm5 (average values across individuals). Due to substantial base deletion and overall transformation, D gene segments were usually unrecognizable without ambiguity. Sequences where Dm could be identified unambiguously and accurately (with minimum length = 4 nucleotide (nt)) represent 816,556 out of the 5,815,283 VJm sequences. For these 816,556 sequences, a similar trend in Dm usage was observed in individual fish, with Dm1 showing the highest frequency (Figure 1A).

**Figure 2:**
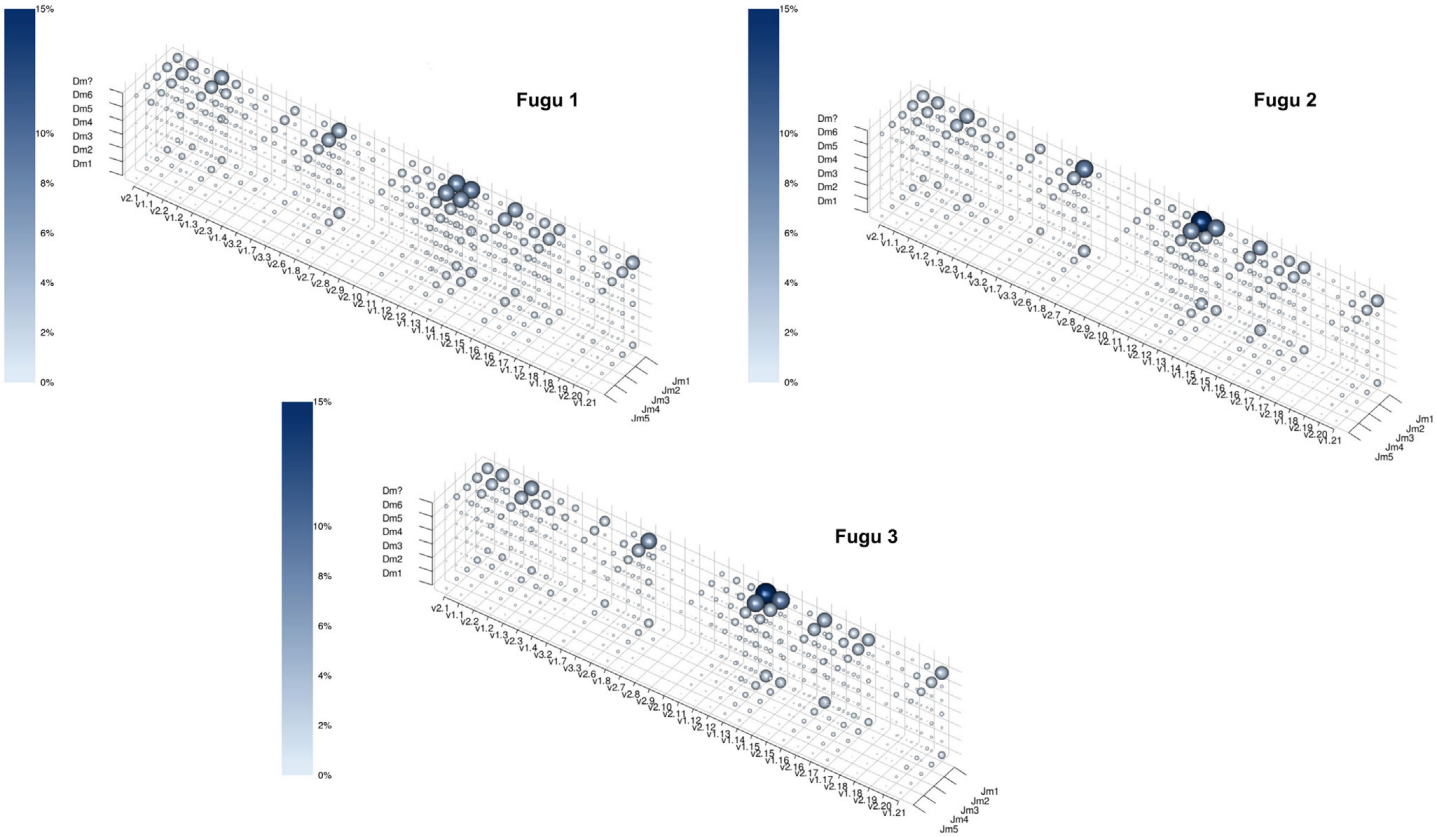
The captured VDJ repertoires for individual torafugu. Each point in the three-dimensional space represents a unique VDJ combination. Both the size of the sphere and the intensity of the color correspond to the percentage of the number of reads that match the VDJ combination relative to all captured reads. To prevent a sphere overlapping with others, the size of the sphere was transformed by *n* ^(1/4)^, where *n* is the percentage of the corresponding VDJ combination. “Dm?” represents ambiguous D segment.

**Figure 3:**
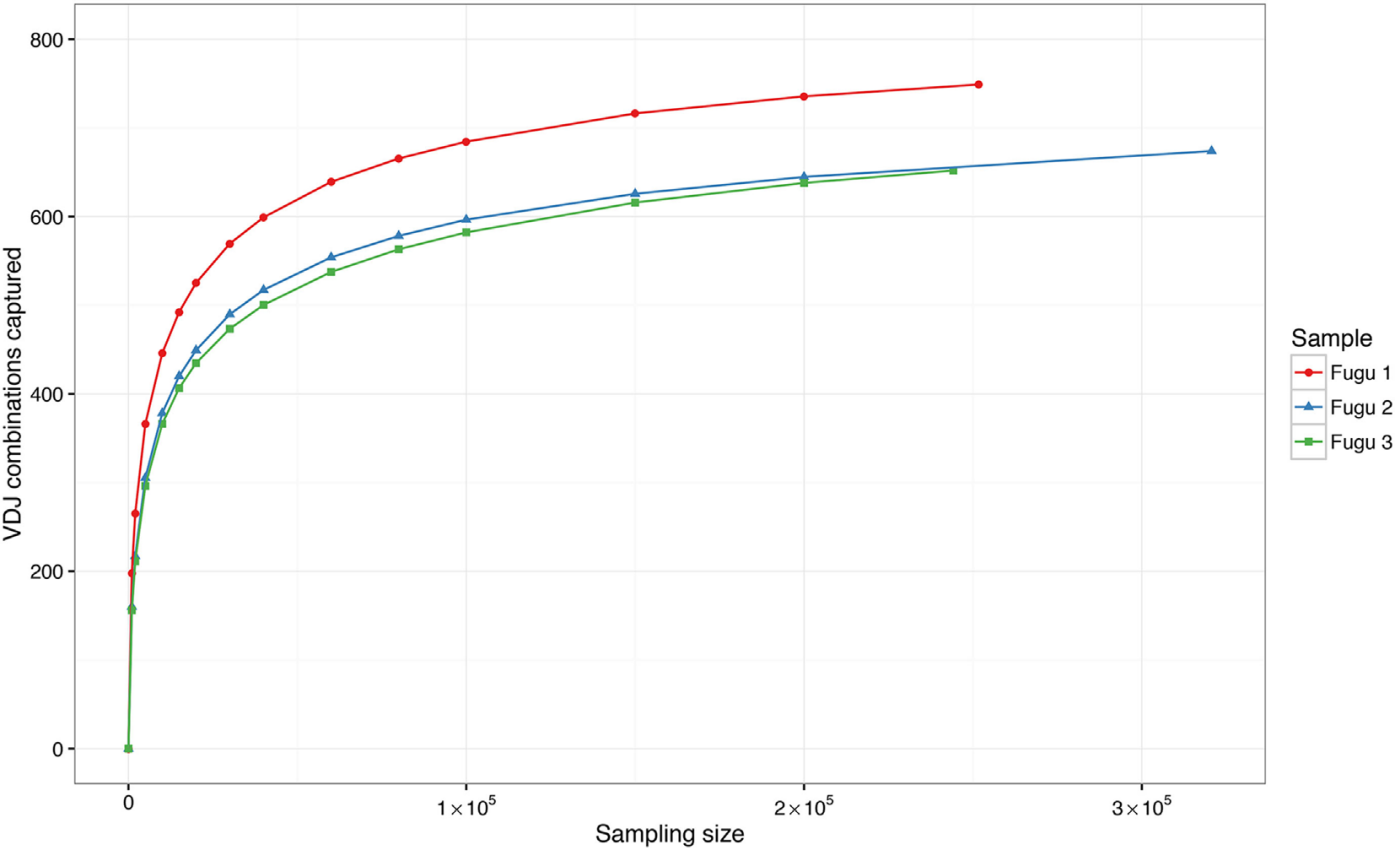
Rarefaction studies of VDJ diversity in IgM. It demonstrates that with deeper sequencing in an individual, the number of novel VDJ combinations saturates. The sampling–resampling process was performed 1,000 times for each data point.

Theoretically, there are 960 possible VDJ combinations in torafugu (32 V × 6 Dm × 5 Jm = 960 VDJ). The collection of all VDJ combinations is deemed as the VDJ repertoire. In the present study, a total of 788 VDJ combinations were captured. Individual torafugu VDJ repertoires could be visualized in 3D graphical form (Figure 2). The combination coverage in any captured torafugu ranges from 70 to 82%, showing similar tendencies compared with the previous observation in zebrafish (8). Most VDJ combinations showed low abundance; however, a similarly small fraction—albeit showing different ranking for specific combinations in individual fish—were observed at high frequencies. Consistently, there was a strong overlap among the top 10 (accounting for an average of over 45% of frequency in all captured combinations) VDJ combinations in individual fish, whereas most combinations were identified only once. We also demonstrated that the sampling of the VDJ repertoire was directed toward saturation by performing rarefaction studies (Figure 3).

#### IgT

IgT rearrangements are independent of IgM and are carried out using distinct sets of D and J gene segments. The observed usage pattern of V gene segments suggests that there is a commonality to the frequency distributions of V in IgM, which is not observed in IgT. As can be noted in Figure 1B, although the expression of IGHV2 family V sequences in IgT was commonly observed, the usage of individual IGHV2 gene segment was much more diverse across fish than those in IgM.

Differential expression of IgM and IgT has been documented in torafugu (17), and there is some knowledge about their tissue-specific expression as well as their developmental progression; IgM expresses as early as 4 days’ post fertilization whereas secretory IgT is observed 4 days later. The finding that V(DJ)-segmental profiles of IgM and IgT are distinct from each other may suggest that the repertoires of IgM- and IgT-secreting B cells are different, and that the two types of B cells bear different distributions of variable regions for antigen binding.

### Sequence Diversity of CDR-H3

Here, we defined the position of CDR-H3 as the region between the last conserved cysteine of the V segment and the first conserved tryptophan of the J segment in the conserved motif, WGxG. In the pooled data set, the standard defined subsets of 3,196,578 and 4,345,245 CDR-H3 aa sequences of IgM and IgT, respectively.

#### IgM

**Figure 4:**
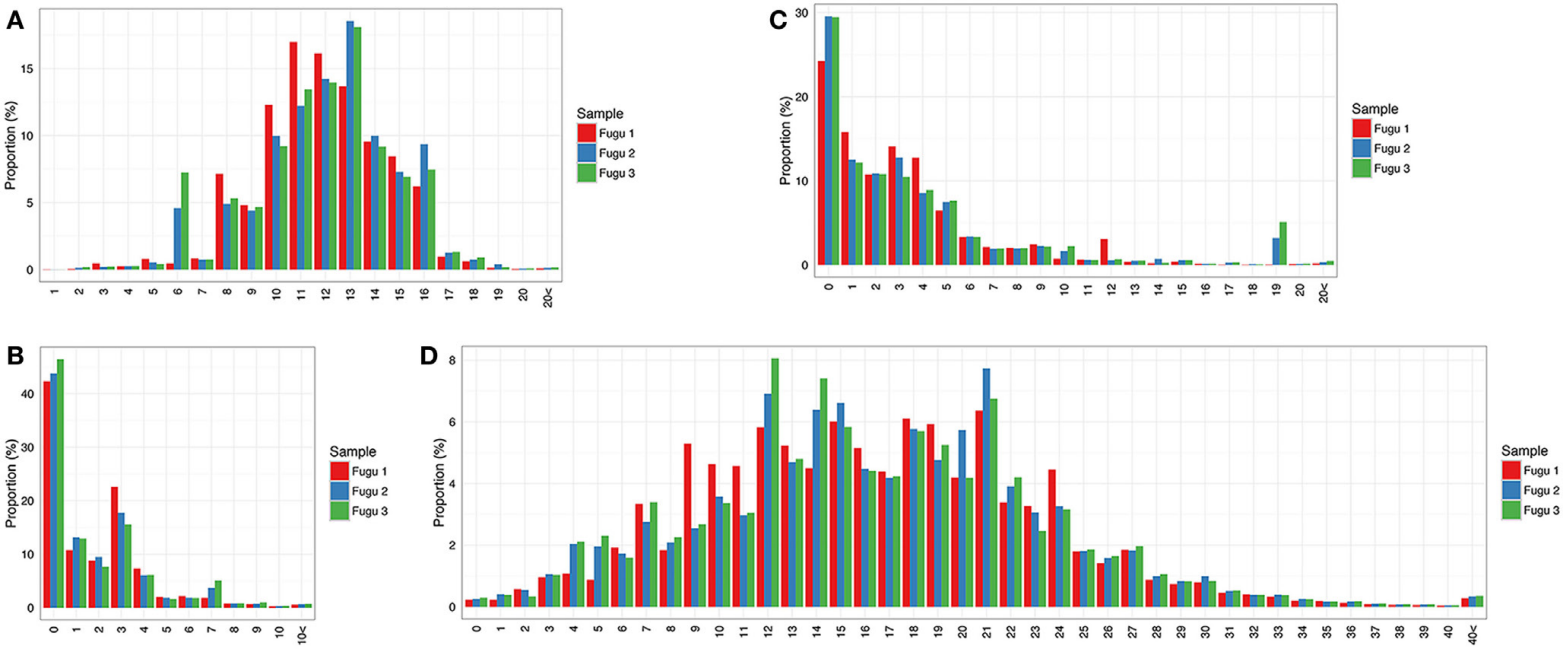
Sequence diversity of heavy (H) chain complementarity-determining region 3 (CDR-H3) repertoire in IgM. **(A)** Distribution of CDR-H3 amino-acid length. **(B,C)** Distribution of the total number of nucleotide deletions and **(D)** V–J insertion.

It was observed that most captured CDR-H3 sequences fall in a length range from 8 to 16 aa with a bell-shaped profile (Figure 4A), which has been documented in other teleosts (i.e., rainbow trout) (12) and is suggested as a representative feature of the immune repertoire expressed by naïve lymphocytes in mammals (18). In addition to the combinatorial variety, most rearrangements involved non-template nt deletion and insertion, the extent of change within CDR-H3 could be substantial. Here, we investigated random deletion of nts and insertion of non-template nts at the V–D and D–J junctions. As a result, much of the observed diversity in the torafugu CDR-H3 repertoire was generated by insertion of non-template nts: (i) more than 80% of the observed sequences had zero or less than three deletions at the V–D (Figure 4B) and D–J junctions for each fish (Figure 4C); (ii) in contrast, most sequences with ten or more nt insertions were observed in the region between 3′ V and 5′ J (refers to regions that contain the additional non-template nts along with the remnants of the D gene segment used which cannot be distinguished based on the current method) in individuals (Figure 4D).

In our dataset, there are examples of independent recombination events that have produced the same CDR-H3 aa sequence. An average of 411,984 distinctive CDR-H3 aa sequences for each fish were captured. Although most captured CDR-H3 aa sequences were encoded by only 1 nt sequence, we found on an average 37,190 examples of rearrangements, constituting approximately 10% of the total, that the same CDR-H3 aa sequence could be encoded by divergent nt sequences (Table 2). Additionally, in the mean 43,411 instances, the same CDR-H3 nt sequence was associated with different VJ pairing sequences.

**Table 2:** Summary of the convergent recombination of nucleotide (nt) sequences for heavy (H) chain complementarity-determining region 3 (CDR-H3) amino-acid (aa) sequences in IgM and IgT.

	Fish	1	2	3
IgM	Unique CDR-H3 aa sequences	363,728	479,043	393,180
	CDR-H3 aa with 1 coding sequence	330,174	435,994	358,213
	CDR-H3 aa with >1 coding sequences	33,554	43,049	34,967
	Ratio of CDR-H3 aa with >1 representative nt sequences	0.092	0.089	0.088

IgT	Unique CDR-H3 aa sequences	93,683	72,364	90,292
	CDR-H3 aa with 1 coding sequence	78,573	60,121	76,454
	CDR-H3 aa with >1 coding sequences	15,110	12,243	13,838
	Ratio of CDR-H3 aa with >1 representative nt sequences	0.161	0.169	0.153

#### IgT

**Figure 5:**
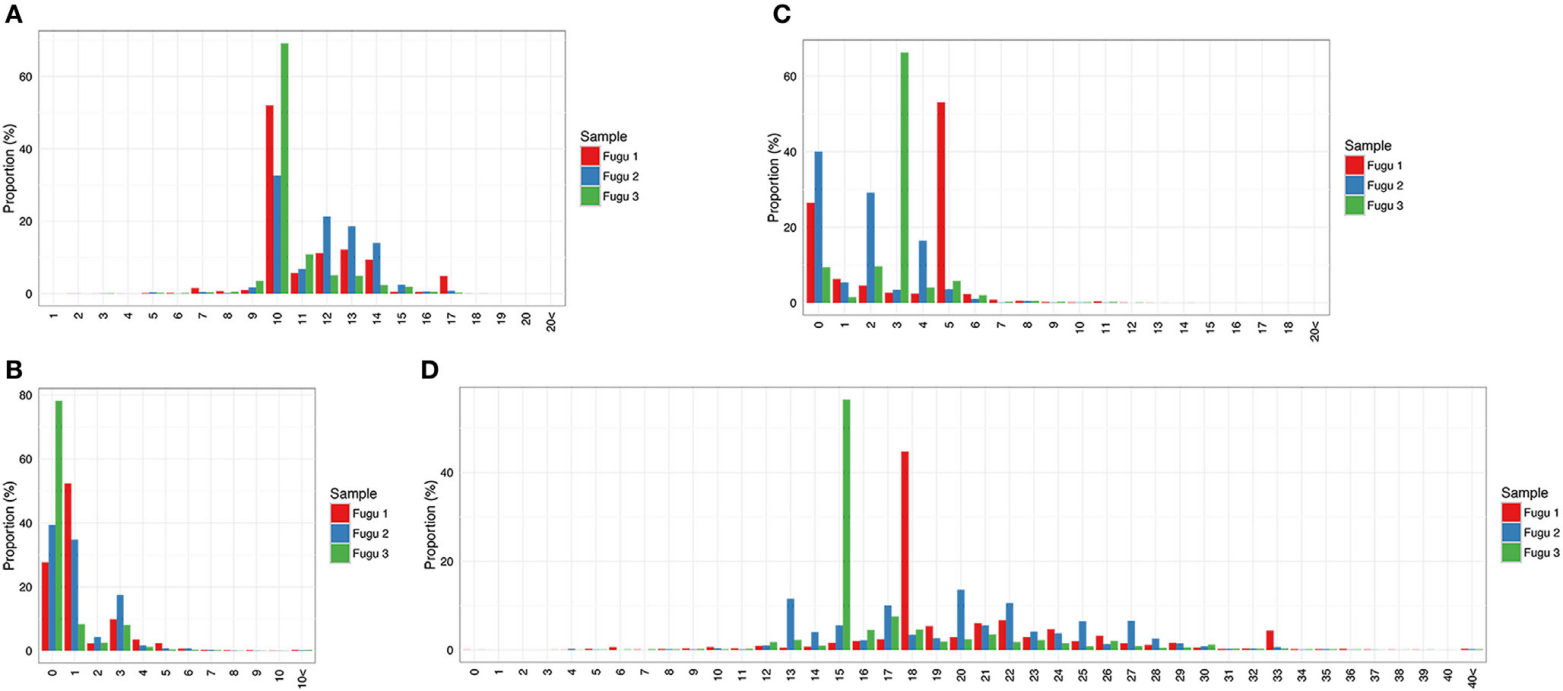
Sequence diversity of heavy (H) chain complementarity-determining region 3 (CDR-H3) repertoire in IgT. **(A)** Distribution of CDR-H3 amino-acid length. **(B,C)** Distribution of the total number of nucleotide deletions and **(D)** V–J insertion.

Compared with IgM, IgT had a narrower distribution of CDR-H3 region lengths, assuming a fewer number of peaks (Figure 5A). Specifically, the CDR-H3 length of IgT varied from 7 to 17 aa with a peak at 10 aa. Like IgM, most CDR-H3 diversity in IgT was generated by insertion of nts. However, the distribution frequency of junctional diversity in IgT differed from that in IgM. For IgM, the non-random base addition/deletion at the V–D and D–J junctions was apparent: insertion/deletion was observed at a roughly equal frequency in the three individuals. However, no clear trend was seen for IgT; junction size showed few localized peaks (Figures 5B–D); a skewed and restricted insertion/deletion dominating each of the three individuals, i.e., fugu 1 had the highest frequency (45%) of 18 insertions at the two junctions whereas fugu 3 had 15 insertions with the highest frequency (56%) (Figure 5D).

The captured distinct CDR-H3 aa sequences in IgT were much fewer than in IgM and similar nt–aa usage trend was observed for IgT. About 15% of the total CDR-H3 aa sequences were from more than 1 nt sequence, whereas most of them were exclusively encoded by 1 nt sequence (Table 2). On an average, there were 14,577 instances wherein each CDR-H3 nt sequence could be associated with different (more than one) VJ pairings.

### Estimating CDR-H3 Repertoire Size

The vast diversity of CDR-H3 is essential for maintaining functional immune responses. To explore this, we performed a capture–recapture analysis to estimate the potential size of the CDR-H3 repertoire from our sequence data. This approach is commonly used for estimating population sizes and diversity in general (19) or in immunological studies (20, 21). We first combined similar yet slightly distinct CDR-H3 aa sequences differing by at most 20% due to mutation and sequencing/PCR errors, to a single CDR-H3 cluster. We then applied the sample and resample technique on the three individuals to estimate the size of the torafugu CDR-H3 repertoires. This analysis yielded an estimate of, 503,559 IgM and 76,344 IgT lineages with pooled data (Table 3). However, since the pooled data has a large number of clusters that contain only one unique sequence (Figure S1 in Supplementary Material), the number of torafugu Ig lineages might be overestimated, as suggested by Haegeman et al. (22). Comparison of the actual size of observed CDR-H3 clusters with the estimated ones revealed that 74% IgM and 78% IgT of the total CDR-H3 clusters were captured in our dataset (Table 3). Because the effect of VDJ combination diversity on CDR-H3 aa diversity is not trivial, we also calculated the average ratio between the number of CDR-H3 aa clusters and the number of captured VDJ combinations in IgM (503,559 clusters/788 VDJ = 639) and IgT (76,344 clusters/30 VDJ = 2,545), respectively. Such an observation might implicate a higher degree of repertoire diversity in naïve IgT^+^ B cells than in IgM^+^ B cells.

**Table 3:** Heavy (H) chain complementarity-determining region 3 (CDR-H3) amino-acid (aa) clustering statistics.

	IgM			IgT		
Fish	CDR-H3 aa	CDR-H3 aa clusters^a^	Population size^b^	CDR-H3 aa	CDR-H3 aa clusters	Population size
1	929,522	156,730	230,079	1,663,460	25,931	35,513
2	1,225,559	201,461	292,175	1,177,589	20,920	28,655
3	1,041,497	181,996	280,449	1,504,196	27,949	38,128
Pooled	3,196,578	372,933	503,559	4,345,245	59,619	76,344

*^a^Number of CDR-H3 clusters computed by CD-HIT*.

*^b^Number of population clusters estimated by abundance-based coverage estimator*.

### Commonly Used CDR-H3 Sequences among Individuals

**Figure 6:**
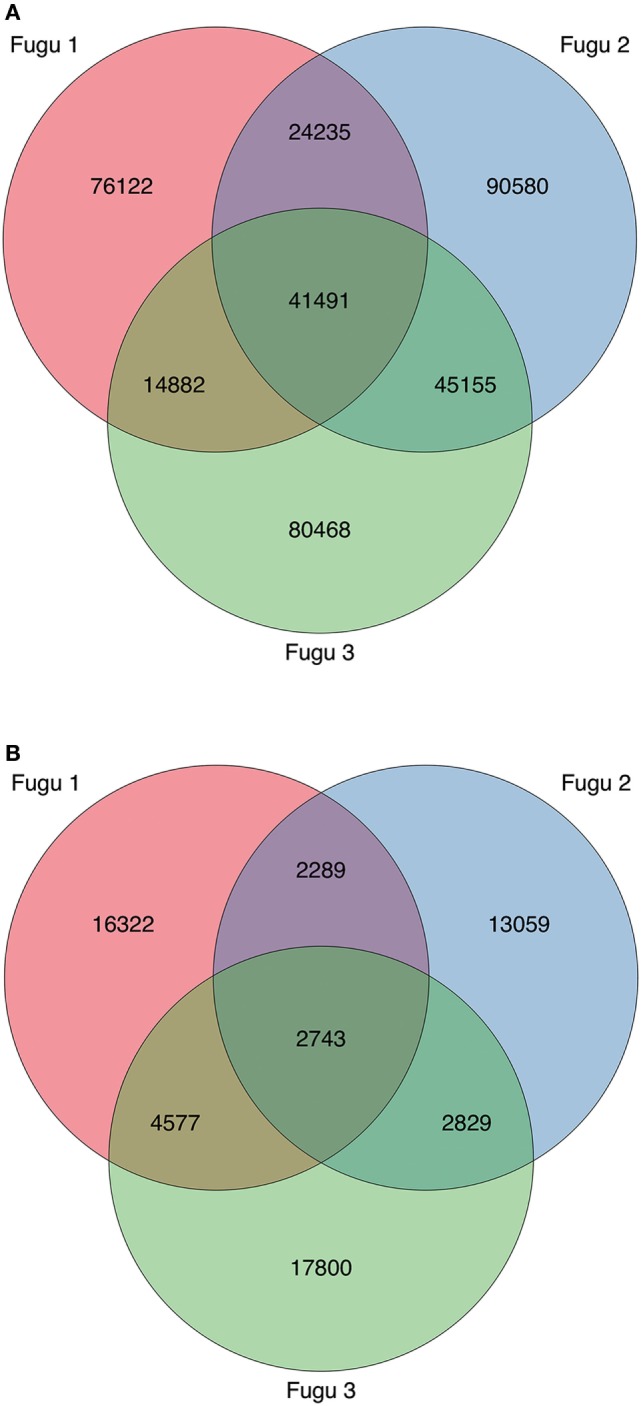
Profile and overlap of heavy (H) chain complementarity-determining region 3 (CDR-H3) cluster abundance across the three torafugu. **(A)** IgM and **(B)** IgT. The Venn diagram corresponds to Table 3 showing the cluster sequence statistics.

**Figure 7:**
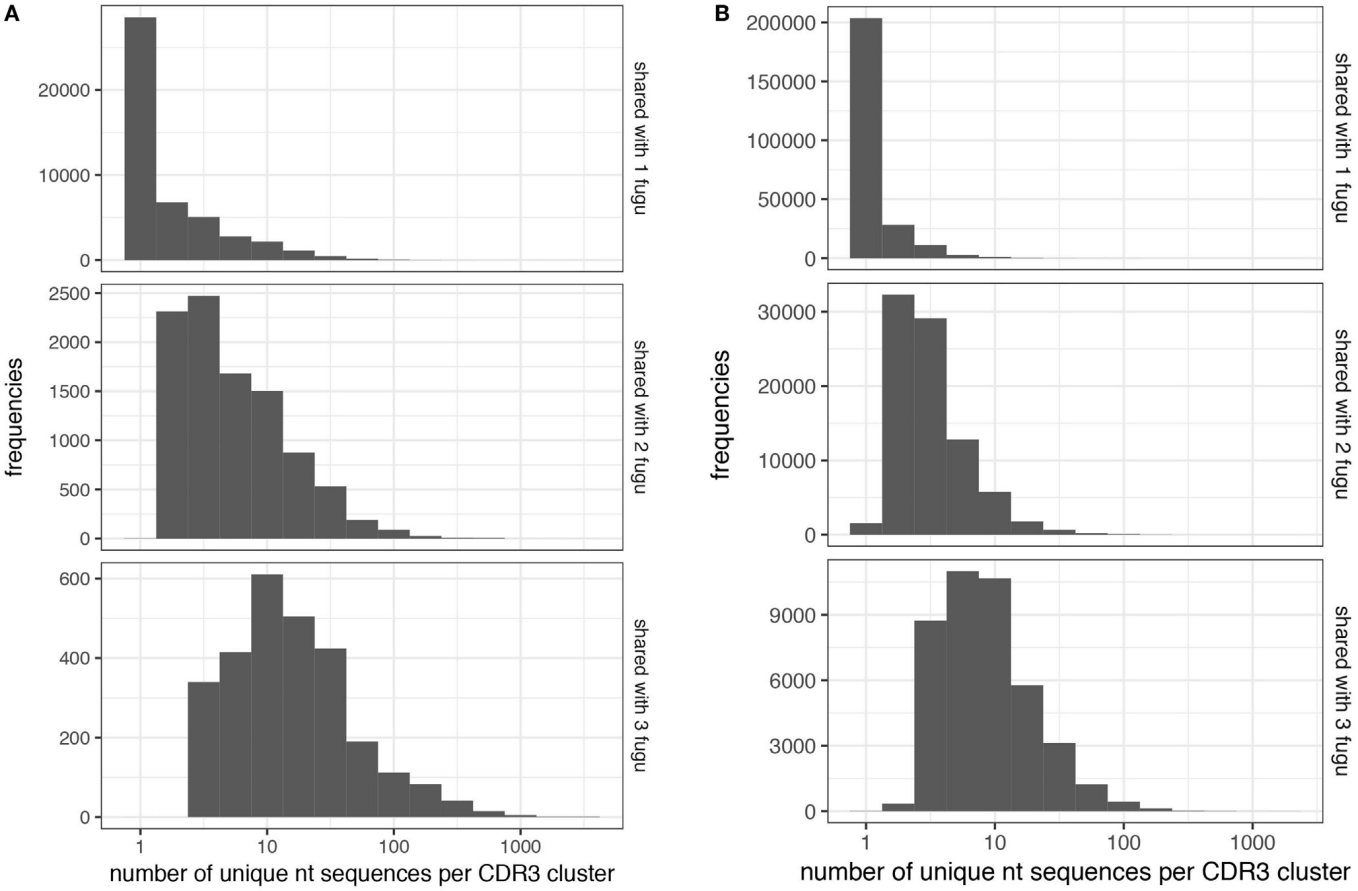
Convergent recombination analysis of heavy (H) chain complementarity-determining region 3 (CDR-H3) clusters. Convergent recombination gradually increases as a function of sharing in **(A)** IgT and **(B)** IgM CDR-H3 clusters.

Next, we explored the CDR-H3 characteristics based on the sharing level (Figures 6A,B). A CDR-H3 cluster was formed with a group of CDR-H3 aa sequences with at least 80% similarities. Our analysis revealed that common CDR-H3 aa clusters (i.e., found in more than one individual) manifest a higher level of “convergent recombination” (the same CDR3 aa sequence could be generated from different nt recombinations) (23, 24); increased sharing was involved with a gradual increase in the mean degree of convergent recombination. CDR-H3 aa clusters found in one individual (termed as “private CDR-H3 cluster”) were encoded by one (the median value) nt sequence for both IgM and IgT, whereas the common CDR-H3 clusters were encoded by 14 (for IgT; Figure 7A) and 8 (for IgM; Figure 7B) nt sequences on average; similar behavior was shown for T cells (25–27). We also compared the contents of sequence abundance, average length, and mutation levels between private and common CDR-H3 aa clusters; however, no significant difference was observed, with similar distribution trend appearing in groups (Figure S2 in Supplementary Material). This may demonstrate that shared CDR-H3 aa sequences (clusters) differ from private ones in a detectable degree of convergent recombination, irrespective of their intrinsic characteristics.

Finally, although CDR-H3 clusters were common among individuals at a low level (8% for IgM and 4% for IgT), 41,491 IgM and 2,743 IgT shared clusters were identified in our dataset, indicating a potential for public responses in this species.

## Materials and Methods

### Torafugu and Total RNA Preparation

Adult torafugu (*n* = 3) weighing between 800 and 900 g were obtained from Fish Interior (Tokyo, Japan). All fish were maintained in tanks with aerated seawater at 20°C. The fish were euthanized, followed by rapid dissection of tissues. Spleens and trunk kidneys from each torafugu were collected and directly fixed in RNA*later*^®^ (Ambion, Austin, TX, USA). Total RNA was extracted using the RNeasy Lipid Tissue Mini Kit (Qiagen, Valencia, CA, USA). The mRNA was further purified using Dynabeads^®^ mRNA DIRECT™ Purification Kit (Ambion, Austin, TX, USA). Manufacturer’s protocols were followed during these processes and the concentrations of both the total RNA and mRNA were measured using a Qubit3.0 fluorometer.

### Primer Design

The torafugu IgH locus was described in our previous study (28) (Figure S3 in Supplementary Material). Here, we used the IgM and IgT classes referring to the protein products of their isotypes, μ and τ, respectively, which correspond to their associated constant region genes. The consensus leader sequences for 32 potentially functional V genes were used to design five forward primers (as part of a family) (Table S1 in Supplementary Material). The reverse primers were derived from the first exon of Cμ and the second exon of Cτ, respectively (Figure S4 in Supplementary Material). To obtain cleaner products, a second independent primer set (Table S1 in Supplementary Material) was designed. The forward primers of Set 2 utilized the consensus frame region 1 (FR1) sequences for each IGHV family, and the reverse primers were based on the nested Cμ and Cτ sequences; these (nVhCm1 and nVhCt1) were located 3-nt upstream from the first round Cμ-specific PCR primer and in the first exon of Cτ. Gene-specific primers (GSP-μ and GSP-τ) were also designed for reverse transcription.

### cDNA Synthesis and Multiplex PCR Amplification

First-strand cDNA was synthesized using SuperScript^®^ III reverse transcriptase (Invitrogen, Carlsbad, CA, USA). Total mRNA purified from each fish was split into four cDNA synthesis reactions with both the primers for IgM and IgT constant regions. RNase H (Invitrogen, Carlsbad, CA, USA) was added to each reaction to remove RNA at the end of the cDNA synthesis step.

Each 20-µL cDNA synthesis reaction was split into two PCRs, and a total of eight PCRs were set up for individual fish. Each of the five forward primers was added to represent each V segment at a final concentration of 300 nM. Some primers covered multiple V segments, and their concentration was proportionate with the number of V segments. Both reverse primers were added at a concentration of 10 µM. The reverse transcription reaction (2 µL) subsequently served as a template for PCR amplification using Platinum^®^ *Taq* DNA Polymerase High Fidelity (Invitrogen, Carlsbad, CA, USA). The thermal cycling conditions were as follows: 94°C for 2 min, followed by 30 cycles of 94°C for 30 s, 55°C for 30 s, and 68°C for 1 min, and a final extension at 68°C for 5 min. PCR products were purified using QIAquick PCR Purification Kit (Qiagen, Valencia, CA, USA). A second-round PCR was performed on 2 µL of the first-round reaction with proportionate nested primers. Reaction conditions were as follows: 94°C for 2 min, 28 cycles of 94°C for 30 s, 55°C for 30 s, and 68°C for 1 min, and a final 5-min extension at 68°C. The second-round PCR products were purified as described above, and their concentration was measured using a Qubit^®^ 3.0 fluorometer. The size distribution of the PCR products was determined using the Agilent 2200 Tape Station.

### Amplicon Library Construction

About 1 µg of QIAquick cleaned PCR product for each fish was used to initiate the Illumina library preparation process. The Illumina TruSeq^®^ DNA PCR-free sample preparation protocol was followed with slight modification. Briefly, double-stranded DNA was end-repaired and ligated to indexing adaptors for hybridization onto a flow cell. The DNA library templates were quantified by qPCR using a 7300 real-time PCR cycler (Applied Biosystems, Waltham, MA, USA) and the Library Quant Illumina Kit (KAPA Biosystems, Boston, MA, USA) with standards in a range from 0.2 fM to 20 pM. Finally, equal amounts of quantified cDNA (10 ng) from each of the three libraries, corresponding to each fish, were pooled to obtain the final amplicon library, which represented the complete collection of IgH transcripts from the three fishes.

### Library Sequencing and Pre-Processing of Data

Libraries were sequenced on the Illumina MiSeq platform using the V3 (2 × 300 base) kit. The sequencing runs yielded approximately 6 million raw reads per sample. Raw reads were sorted into isotypes IgM/IgT according to their primer sequences using cutadapt (29) (parameter: −O 10, −e 0.2), and bases with a Phred quality score *Q* < 20 were trimmed from the 3’-end and the 5’-end by Trimmomatic (30) (parameter: LEADING:20, TRAILING:20, MINLEN:30). The remaining paired reads were merged using PEAR (31) (parameter: −v 10, −p 0.05, −n 300).

### Data Analysis

Most existing tools [e.g., IMGT/V-Quest (32), IgBLAST (33), and JoinSolver (34)] for Ig repertoire analysis are restricted to organisms registered with International ImMunoGeneTics (IMGT). Till now, sequence annotation for torafugu Ig sequences remains unavailable on IMGT. Here, we have developed PyDAIR (https://github.com/biunit/PyDAIR) as a more flexible tool for analyzing Ig sequences. This tool can be applied to any organism without database restriction.

Identification of the VDJ usage and definition of the CDR-H3 sequence were performed using an in-house developed pipeline as illustrated in Figure S5 in Supplementary Material. In brief, merged (consensus) reads were first aligned to each V- and J-germline sequence using the Basic Local Alignment Search Tool (BLAST) to determine the optimal alignment (parameters used are shown in Table S2 in Supplementary Material). The CDR-H3 sequences were then extracted by locating the two conserved motifs (Table S3 in Supplementary Material) between V (the 2nd CYS) (35) and J (the tryptophan in the conserved WGxG motif characteristic of J) (17) using regular expression matching. Finally, parts of the CDR-H3 sequences were aligned to D-germline sequences for complete D identification (Figure S5 in Supplementary Material). We counted the read number of each V, D, and J gene segment and calculated the frequency distribution. The computational strategy used for indel detection within the VDJ junctions was adapted from Decombinator (36).

### CDR-H3 Diversity Estimation

Capture–recapture analysis was performed to assess the CDR-H3 diversity. CDR-H3 aa sequences were clustered into lineages according to sequence similarity using CD-HIT (37, 38). Clusters were created according to the following steps.

First, CDR-H3 sequences from the three samples were pooled. Then, each CDR-H3 sequence in the pool was compared with all other sequences using CD-HIT (parameter: −c 0.8, −n 4, −s 0.8, −M 2000, −l 5, −d 200). Input CDR-H3 sequences were added to the same cluster if they shared at least 80% similarity and that the shorter one matched at least 80% of the length of the longer one. The number of CDR-H3 clusters for each sample and for the pooled data are summarized in Table S6 in Supplementary Material. The population sizes of CDR-H3 repertoires (i.e., CDR-H3 sequence clusters) were then estimated with abundance-based coverage estimator (19).

## Discussion

Here, we characterized the expressed μ and τ IgH repertoires (IgM and IgT) in adult torafugu by massively parallel sequencing of IgH amplicons. The approach developed in this study allowed us to identify Ig sequences and determine the abundance and isotype of IgH mRNA as well as the CDR-H3 diversity. Consistent with previous observations from other groups (8, 11, 39), we observed that certain V, D, and J gene segments were preferentially used in torafugu. This observation highlighted the usage bias of V, D, and J gene segments in the quiescent immune system of torafugu, which reflects bias in the VDJ recombination mechanisms. The reasons for such bias are not well understood but are likely due to a combination of proximity effects that influence BCR development. It is also possible that preferential PCR amplification of certain V sequences over others skewed the usage frequencies presented here.

Notably, there is an obvious connection between J gene segments in the VDJ combinations of the IgM repertoire and their relative positions to V and D gene segments on the H chain locus. J gene segments that are close to the V and D gene segments are preferentially used by adult torafugu. In detail, Jm1 and Jm2 accounted for 55 and 31%, respectively, of the Jm usage compared with an expected frequency of 20% assuming unbiased gene usage. This observation is reminiscent of reports in humans that (*i*) V gene segment that are close to the D and J segments are preferentially used (40, 41) and (*ii*) D genes positioned in the J proximal locus are preferentially used in sterile DJ rearrangements (42). These results suggest that the antibody variable region repertoire is partly determined by the chromosomal position of these gene segments. One explanation for such restriction could be chromatin conformations that render certain gene segments more accessible than others (43). In addition, recent research has suggested that chromatin structure governs a large part of the biases in TCR Jβ gene usage in both mice and humans (44). Since it is a general approach, we speculate that the observed location-related Jm biases could be further explained by this theory.

When studying the IgM repertoire, a high stereotypy, i.e., a biased yet common use of a small number of VDJ combinations, was observed. Similarly, in the large-scale sequencing of the naïve zebrafish IgM repertoire, convergences have been found in the normal repertoire (8). This result suggests the presence of a common pool of pre-existing B cells in which the structured IgM repertoire could be recruited. It is also possible that the source of the naïve IgM repertoire is convergent evolution, and fish living similar environments show that selection in the quiescent immune systems converges to certain VDJ combinations. Finally, the observed public IgM component is consistent with the paradigm of the B-cell clonal selection theory (specific antigen only activates (i.e., selection) its counter-specific cell to produce its clones for antibody production) as noted in mammals (45, 46), suggesting that general features are already in place in the common ancestors of fish and tetrapod.

We also implemented VDJ junction and mutation analysis as a function of CDR-H3 diversity among different individuals. Our results suggest that most of the junction mutations in adult fish are generated from insertions of nts rather than deletions. The high frequency of nt insertions may correspond to an ordered expression of terminal deoxyribonucleotidyl transferase, as previously noted (47). Interestingly, our analysis of the spread of insertion diversities indicates that the distribution of nt insertion in IgM is much wider than that in IgT. Perhaps this diversity is related to the compartmentalization of the two Ig isotypes.

To observe how often repertoires converged to the same core region of the antibody, we searched for CDR-H3 clusters that are shared between fish. As a result, there were a number of public components consisting of slightly varying CDR-H3 aa sequences that were shared by different individuals. Importantly, this represents a large potential for public responses. Additionally, the same CDR-H3 aa sequence could be encoded by divergent nt sequences. Taken together, these data suggest the powerful forces of antigen-driven clonal selection.

By combining high-throughput sequencing with informatics analysis, we revealed the naïve μ and τ IgH repertoires in torafugu. We discovered that VDJ usage is not uniform and that the same handful VDJ combinations often common to different individuals in the naïve IgM repertoire. We also found that the mutation content of IgM and IgT repertoires is diverse, and that convergent evolution of CDR-H3 sequences is common. Collectively, these data provide a window into the mechanism of Ig diversity creation and allow us to better understand B-cell clonal selection.

## Data Access

The sequencing data from this study have been submitted to DDBJ (http://www.ddbj.nig.ac.jp/) under the accession numbers DRA004021 to DRA004023.

## Ethics Statement

This study was conducted in accordance with the recommendations of “Guidelines for Proper Conduct of Animal Experiments” released by the Science Council of Japan. The protocol was approved by the Subcommittee on Institutional Animal Care and Use of the Graduate School of Agricultural and Life Sciences, The University of Tokyo (Protocol no. P14-952).

## Author Contributions

XF performed experiments, analyzed data, and wrote the manuscript. JS contributed analytic tools and analyzed data. ET performed experiments. KS contributed analytic tools. MR assisted in experiments. SW conceived ideas and oversaw the research. SA conceived ideas, oversaw the research, and cowrote the manuscript. All authors contributed to the manuscript preparation.

## Conflict of Interest Statement

The authors declare that the research was conducted in the absence of any commercial or financial relationships that could be construed as a potential conflict of interest.
