# Supplementary material for: High-Throughput Sequencing of the Expressed Torafugu (Takifugu rubripes) Antibody Sequences Distinguishes IgM and IgT Repertoires and Reveals Evidence of Convergent Evolution
Source: Front Immunol. 2018 Feb 21;9:251. doi: 10.3389/fimmu.2018.00251 (PMC5826340; doi:10.3389/fimmu.2018.00251)
Supplement: Supplementary file 2 [file Presentation_1.PDF]

Supplementary Materials

for

High-throughput sequencing of the expressed torafugu (*Takifugu rubripes*) antibody sequences distinguishes IgM and IgT repertoires and reveals evidence of convergent evolution

Xi Fu[1,2†], Jianqiang Sun[3†], Engkong Tan[2], Kentaro Shimizu[3], Md. Shaheed Reza[4,5], Shugo Watabe[4], Shuichi Asakawa[2*]

[1]*State Key Laboratory of Biotherapy, West China Hospital, Sichuan University and Collaborative Innovation Center, Chengdu, China*

[2]*Laboratory of Aquatic Molecular Biology and Biotechnology, Department of Aquatic Bioscience, Graduate School of Agricultural and Life Sciences, The University of Tokyo, Tokyo, Japan*

[3]*Bioinformational Engineering Laboratory, Department of Biotechnology, Graduate School of Agricultural and Life Sciences, The University of Tokyo, Tokyo, Japan*

[4]*School of Marine Bioscience, Kitasato University, Sagamihara, Japan*

[5]*Department of Fisheries Technology, Bangladesh Agricultural University, Mymensingh, Bangladesh*

[†] **These authors have contributed equally to this work.**

[*]**Correspondence:** Prof. Shuichi Asakawa, The University of Tokyo, asakawa@mail.ecc.u-tokyo.ac.jp

Methods

Detailed description of the method used for data filtering and analysis

Here, PyDAIR was used for identifying the VDJ gene segments and CDR-H3 region, and for detecting indels among the VDJ junctions.

Step 1: V and J assignment

All sequences were aligned using BLAST (i.e, blastn on NCBI BLAST+) (1) against the torafugu V- and J- gene segment databases (Supplementary Dataset) with a match gain of +3, mismatch cost of -3, gap-open penalty of 6, and gap-extend penalty of 6 (Supplementary Table 2). Optimal alignments for V- (V-aligned region) and J- (J-aligned region) gene segments were assigned for each IgH sequence. Only sequences with determined V- and J- assignments were used for downstream analysis.

Step 2: CDR-H3 determination

CDR-H3 is defined as a region between the conserved residues of V (C in 2nd-CYS motif) and J (W in WGxG motif of J) (2–4). Each IgH sequence was translated into the amino acid (aa) sequence, and was searched for the two motifs using regular expression matching. In detail, the YYC motif was sought from the 5′ end of the J-aligned region in the 3′ to 5′ direction and the WGxG motif was searched from the 3′ end of the V-aligned region in the 5′ to 3′ direction, as illustrated in Supplementary Figure 5. Notably, the motif searches were executed separately, and iteration was performed for three possible reading frames, one at a time. It is therefore possible that the reading frame of YYC differs from that of WGxG, resulting in *out-of-frame* CDR-H3 nucleotide (nt) sequences (the sequence length is not a multiple of three). In this case, the CDR-H3 aa sequence was defined as the sequence translated from the first nucleotide of the CDR-H3 nt sequence. After translation, only CDR3-H aa sequences without stop codons were used for downstream CDR-H3 diversity analysis.

Step 3: Indel detection

The computational strategy used for indel detection within the VDJ junctions was adapted from Decombinator (5). Once the V- or J- aligned region is assigned (*local* alignment), PyDAIR adds expected nucleotides of the V or J gene sequence adjacent to the *local* alignment to form a *global* alignment (Supplementary Figure 5C). The number of deletions was calculated by counting bases starting from the end of the last three consecutive nucleotides within the *global* alignment, finishing at the last nucleotide of the

expected V or J gene. Finally, the sequence found between the 3′ end of V and the 5′ end of J was defined as a non-template V-J insertion region.

Step 4: D assignment

The V-J insertion region contains the D gene segment used. PyDAIR employs the V-J insertion region for aligning with the torafugu D gene database (Supplementary Dataset). A V-J insertion sequence was retained if it contained at least 4 nucleotides for D gene identification.

Detailed description of the method used for evaluating PyDAIR performance using artificial torafugu IgH sequences.

In some cases, BLAST can give weak results when the local database consists of highly similar sequences. At this point, we performed a simulation study for evaluating the performance of PyDAIR with torafugu IgH sequences. In total, 10,000 artificial IgM sequences were generated according to the following steps: (*i*) individual V, D, and J gene segment (sequences used for creating BLAST databases) was randomly sampled from genes of the IgM group; (*ii*) nucleotides were randomly trimmed from the 5′- and 3′-end of V, D, and J genes (Supplementary Table 4); (*iii*) nucleotides were randomly inserted into V-D and D-J junctions (Supplementary Table 4); and (*iv*) mutations were generated for each nucleotide throughout the sequence with a probability of 0.05. Probabilities of mutation (substitution or deletion) are set to equal the frequency (i.e., 0.2 for both deletion and each A, T, C, G in substitution). Similar simulation procedures were applied to IgT sequences. Taken together, these results indicate that PyDAIR gave the correct VDJ identification with a correction rate of more than 99.5% in IgM and 99.4% in IgT (Supplementary Table 5).

Supporting Figures and Tables

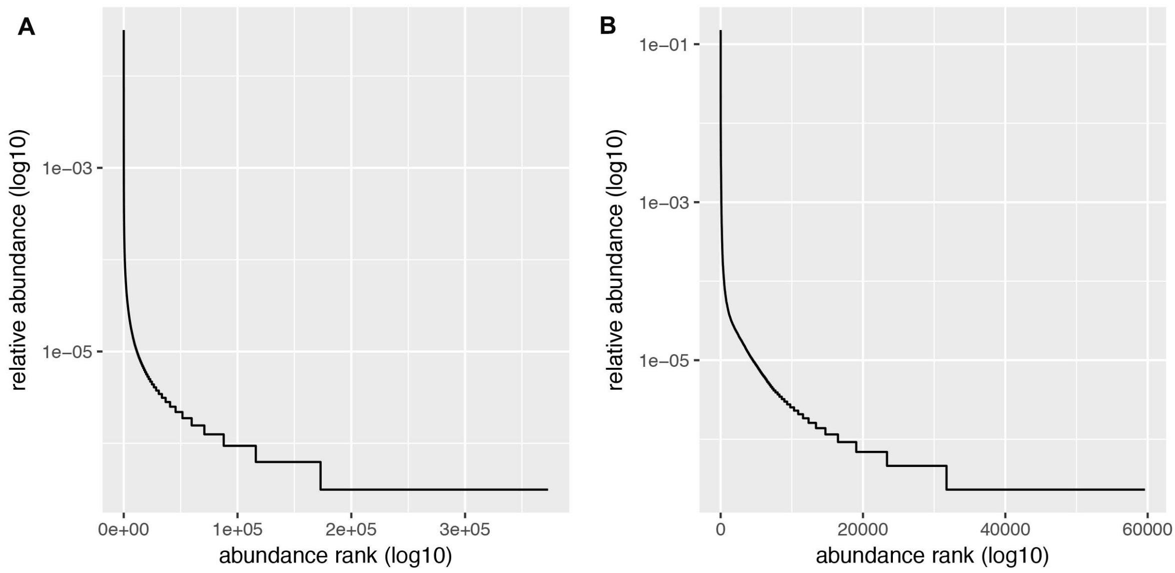

Supplementary Figure 1. Rank-abundance curves representing richness and evenness of CDR3 aa cluster in IgM (A) and IgT (B) groups. X-axis: The abundance rank. The number of individuals in each cluster is sorted in descending order. Y-axis: The proportion of the total number of individuals for each cluster. Both X and Y are plotted by logarithmic scale.

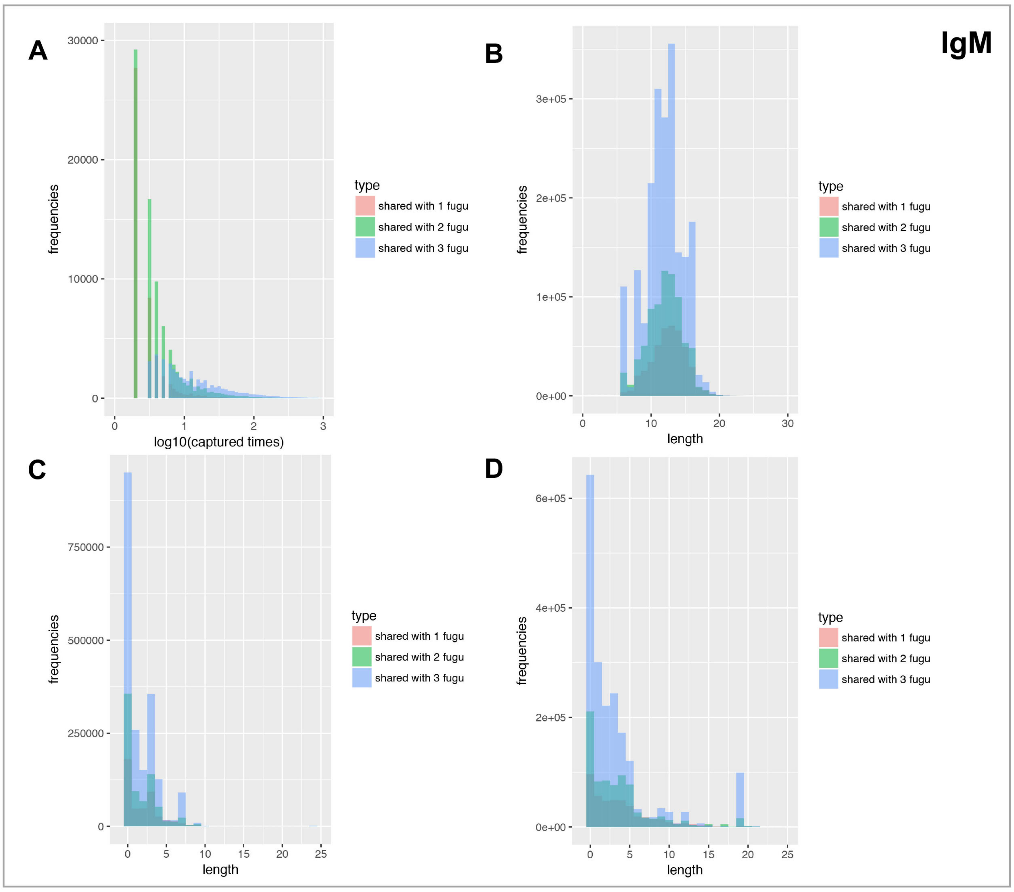
IgM
A
B
C
D
frequencies
log10(captured times)
length
type
shared with 1 fugu
shared with 2 fugu
shared with 3 fugu

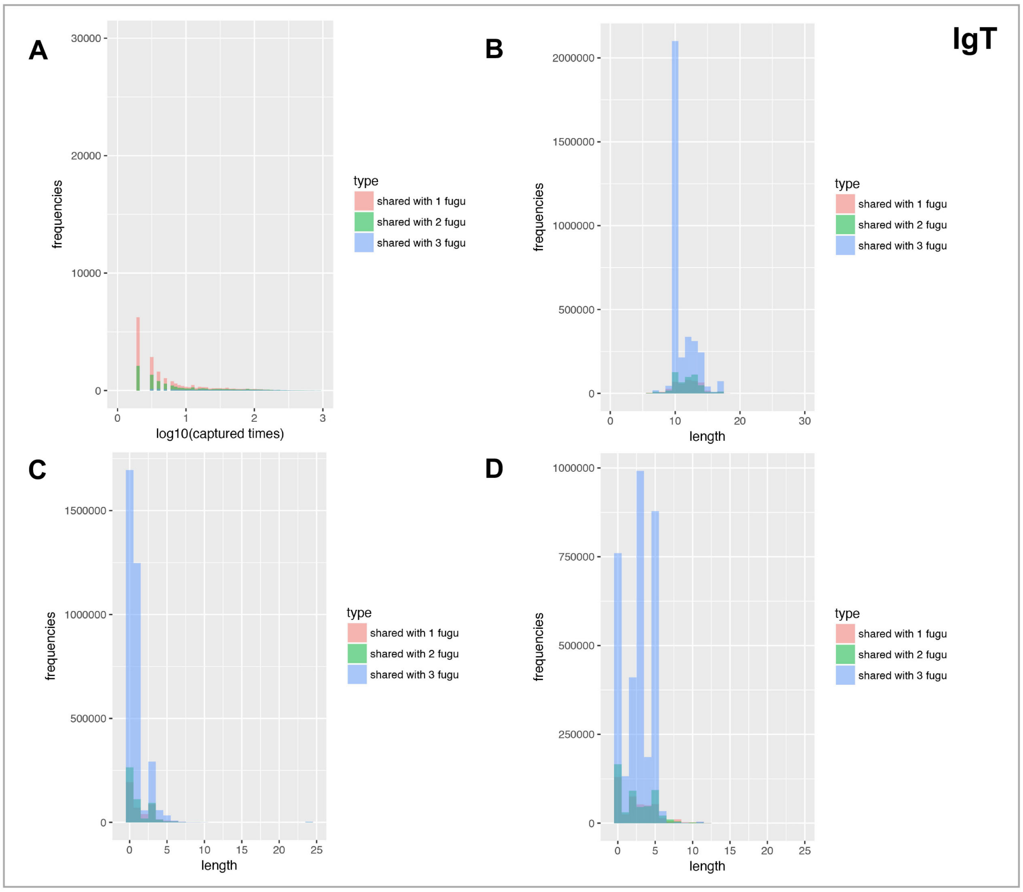
IgT
A
B
C
D
frequencies
log10(captured times)
length
type
shared with 1 fugu
shared with 2 fugu
shared with 3 fugu

Supplementary Figure 2. Characteristics of CDR-H3 sequences shared between individuals. **(A)** CDR-H3 aa sequence abundance distribution. **(B)** CDR-H3 aa sequence length distribution. **(C)** Junctional V-D region length distribution. **(D)** Junctional D-J region length distribution.

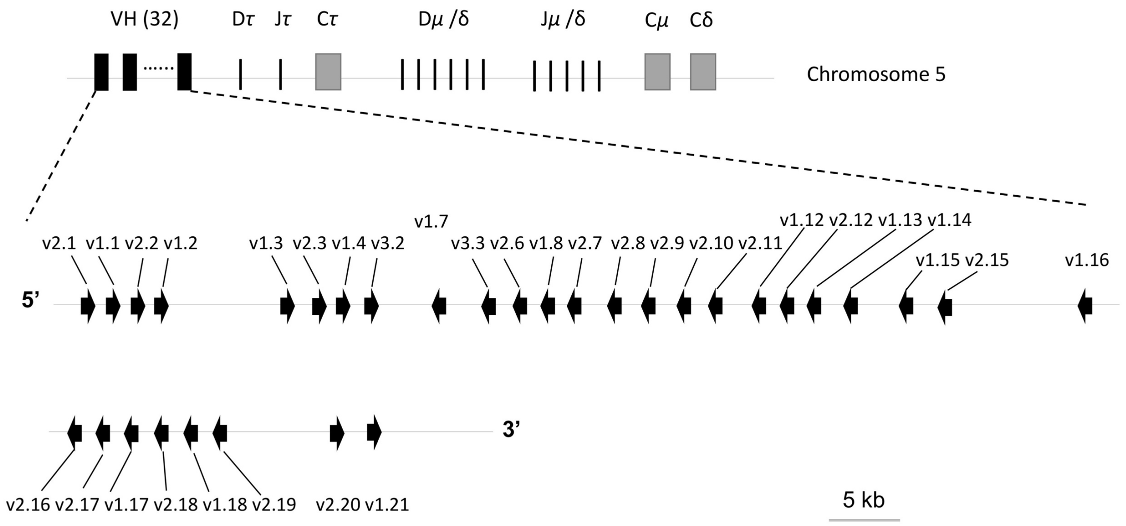

Supplementary Figure 3. Genomic organization of the torafugu Ig H chain locus. The locus is in a 115 kilo-base (kb) region on chromosome 5. The VH region contains 32 potential functional V gene segments and is described in detail (*bottom*), to scale.

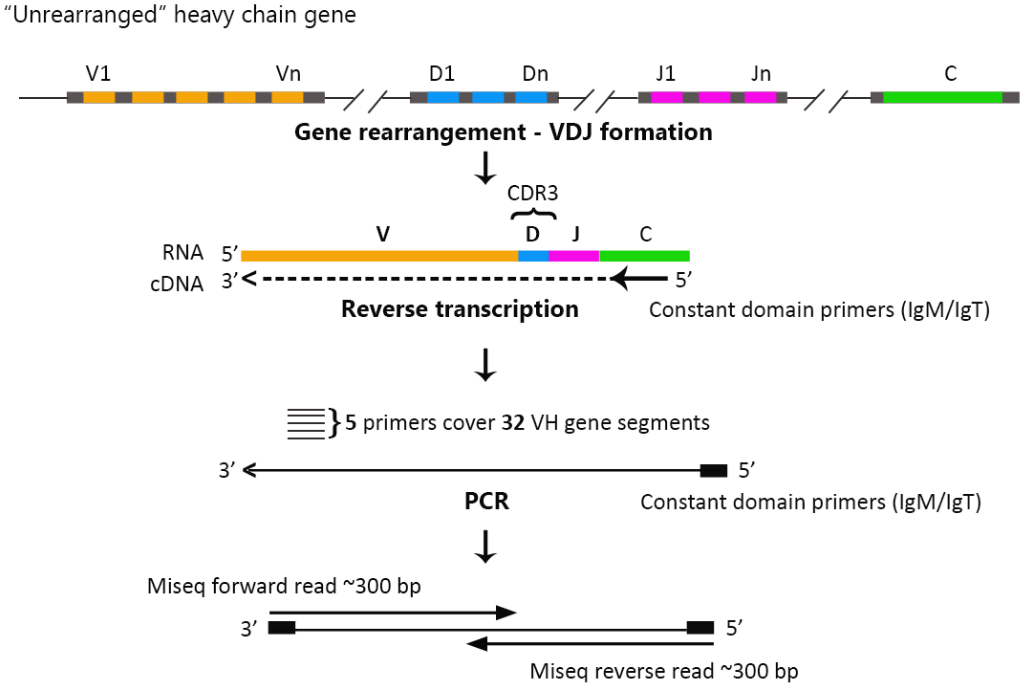

Supplementary Figure 4. Schematic representation of VDJ recombination of the immunoglobulin heavy chain gene, and cDNA amplicon library construction. Primers were designed based on the IGHV family (forward) and the constant region of the IgM/IgT isotype (reverse).

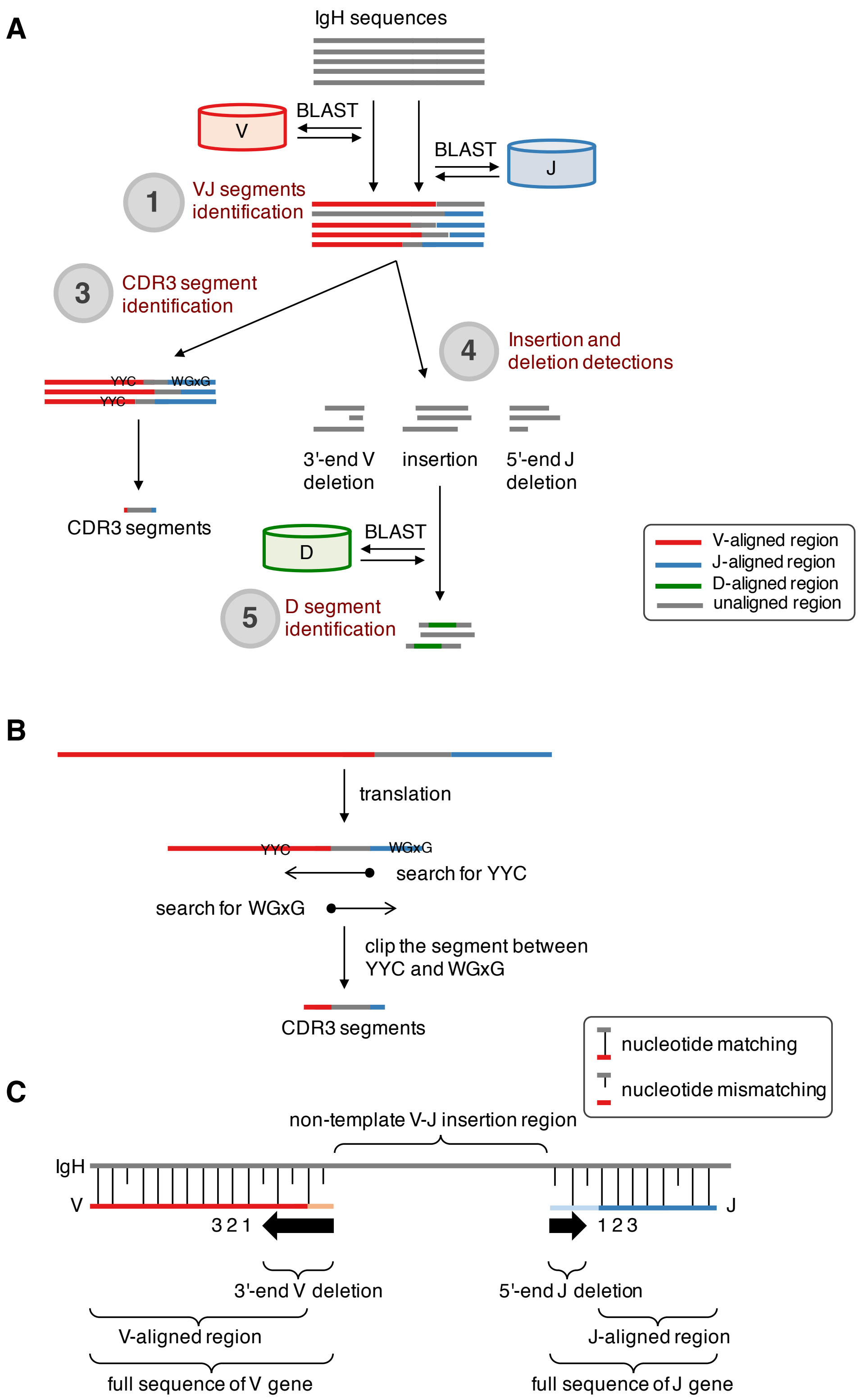

Supplementary Figure 5. Schematic representation of PyDAIR's approach for characterizing IgH sequences. **(A)** Overview of PyDAIR's functions for analyzing IgH sequences including VDJ and CDR-H3 identifications and indel detections. **(B)** Strategy used for determining CDR-H3 sequences. The YYC and WGxG motifs are torafugu-specific. **(C)** Strategy used for detecting indels within V-D and D-J junctions.

Supplementary Table 1. Primers used for cDNA synthesis and multiplex PCR amplification.

Primer	Name	Name of Ig V/C genes	Primer Sequence
Set 1	**FVH1**	IGHV1S1 to IGHV1S4; IGHV1S7; IGHV1S8; IGHV1S12 to IGHV1S18; IGHV1S21	GGACAGGACTGCTGCTTCTAAC
	**FVH2-1**	IGHV2S1; IGHV2S2; IGHV2S6 to IGHV2S12; IGHV2S15 to IGHV2S17; IGHV2S18; IGHV2S20	AGCTCTGCTGCTGCTGTTG
	**FVH2-2**	IGHV2S19	TTCTCTGCAGCTGTGGTGC
	**FVH3-1**	IGHV3S2	CAGAGGTTTACTGATCATTGTC
	**FVH3-2**	IGHV3S3orf	TCTTCAGTGTCTGGTGGACG
	**GSP-μ**	C-μ	AGGGCTACCGTCCCAGTCCTGT
	**GSP-τ**	C-τ	GTGATCAGACACACAAGAGTGACG
	**VhCm1**	C-μ	TGCCGTTCATGGTTGGAGGGT
	**VhCt2**	C-τ	GCTGATCATGTCTTTCTCTGGCG
Set 2	**nFVH1**	IGHV1S1 to IGHV1S4; IGHV1S7; IGHV1S8; IGHV1S12 to IGHV1S18; IGHV1S21	CTGACCCAGTCTGAACCAGT
	**nFVH2**	IGHV2S1; IGHV2S2; IGHV2S6 to IGHV2S12; IGHV2S15 to IGHV2S20	TGAACAGTTGACACAGCCAGC
	**nFVH3**	IGHV3S2; IGHV3S3orf	GCCTGAAGTAAAAGACCTGGA
	**nVhCm1**	C-μ	CGTTCATGGTTGGAGGGTAC
	**nVhCt1**	C-τ	TCTGGGAAGAAGTCGAGAGC

Supplementary Table 2. BLAST parameters used for determining each V, D, and J gene segment.

Gene segment	Match score	Mismatch score	Gap open penalty	Gap extend penalty	Word size	*E*-value
V	3	-3	6	6	10	1e-90
D	1	-1	0	2	4	1e-02
J	3	-3	6	6	7	1e-09

Supplementary Table 3. Motifs used for identifying the CDR-H3 sequence.

Motif name	Motif sequences
2nd-CYS	FFC, YYC, FYC
WGxG	WG.G*

* '.' represents any amino acid

Supplementary Table 4. Default parameters for trimming or inserting boundary bases in the simulation study.

	deletions		additions
tV5	5	aVD	5
tV3	3	aDJ	5
tD5	2		
tD3	2		
tJ5	3		
tJ3	5		

t trimming, a insertion

Supplementary Table 5. Generation of artificial torafugu IgH repertoires and statistical evaluation.

Isotype	Gene segment	Correct	Incorrect	Unidentifiable	Total
	V	9,642	5	353	10,000
**IgM**	D	4,476	2	5,522	10,000
	J	9,646	1	353	10,000
	V	9,422	6	572	10,000
**IgT**	D	6,760	0	3,240	10,000
	J	9,428	0	572	10,000

Supplementary Table 6. Distinct CDR-H3 clusters from three torafugu measured for both IgM and IgT.

Fish	IgM	IgT
Fugu 1	156,730	25,931
Fugu 2	201,461	20,920
Fugu 3	181,996	27,949
Total	540,187	74,800

Supplemental References:

1. F.Altschul S, Gish W, Miller W, Myers EW, Lipman DJ. Basic local alignment search tool. *J Mol Biol* (1990) **215**:403–410. doi:10.1016/S0022-2836(05)80360-2
2. North B, Lehmann A, Dunbrack RL. A new clustering of antibody CDR loop conformations. *J Mol Biol* (2011) **406**:228–256. doi:10.1016/j.jmb.2010.10.030
3. Giudicelli V, Duroux P, Ginestoux C, Folch G, Jabado-Michaloud J, Chaume D, Lefranc M-P. IMGT/LIGM-DB, the IMGT® comprehensive database of immunoglobulin and T cell receptor nucleotide sequences. *Nucleic Acids Res* (2006) **34**:D781–D784. doi:10.1093/nar/gkj088
4. Savan R, Aman A, Sato K, Yamaguchi R, Sakai M. Discovery of a new class of immunoglobulin heavy chain from fugu. *Eur J Immunol* (2005) **35**:3320–3331. doi:10.1002/eji.200535248
5. Thomas N, Heather J, Ndifon W, Shawe-Taylor J, Chain B. Decombinator: a tool for fast, efficient gene assignment in T-cell receptor sequences using a finite state machine. *Bioinformatics* (2013) **29**:542–550. doi:10.1093/bioinformatics/btt004
